# The After-Care of Consumptives

**Published:** 1917-09-29

**Authors:** 


### THE AFTER-CARE OF CONSUMPTIVES.

### The Economy of Developing Existing Institutions.

A feature of tuberculous disease which cannot be missed by anyone who has to do with it for long is a strong tendency to relapse and to recrudescence; what, to borrow a term from criminology, might be called "recidivism." Recidivism is common even in tuberculosis of the lymphatic glands, which is particularly the juvenile form of tuberculosis; more so in tubercle of bones and joints; and most of all, unfortunately, in the great preponderating variety of the disease—namely, pulmonary consumption. Very often this backsliding habit of apparently recovered consumptives must be due to relaxing of treatment brought about by economic necessity, because the current labour market is naturally constituted to meet the requirements of the community rather than of the sick individual. "Light outdoor work," that carelessly given prescription, is hard to get; there are plenty of semi-disabled applicants for it besides the tuberculous contingent. Moreover, when got it is badly paid, and the pay will not go far in any times, especially these, towards the maintenance of a wife and family. Accordingly, efforts have been made, such as at the farm colony near Edinburgh (which, however, does not pay its patients wages), as it were, to temper the wind of competitive livelihood-seeking to the individual shorn by tuberculosis of his power of resistance to adverse conditions. The movement may be seen in all advanced countries. We recently noticed such an institution near Bordeaux, and there is also one in the vicinity of Hanover; at this last the stay is short (two months), but work is paid for at the rate of about three-halfpence an hour. The claims of soldiers invalided for tuberculosis, who, to our sorrow, will form a huge class in a very short time, must stimulate the philanthropic efforts under notice. Indeed it is said that in Worcestershire such provision is to be made on a fairly large scale. In a recent report the Leicester medical officer of health gives an account of a little experiment in employing consumptives in gardens belonging to the Corporation.

Probably the most economical way to get to work—and if that was really the only way for the anti-tuberculosis campaigner before the war, it is a hundred times more so now—will be first of all to develop the capacities of existing sanatoria in the needed direction, rather than to launch out into the establishing of many fresh farm colony institutions. Nearly every sanatorium has some land round it, some of them a considerable amount. Probably shrubberies, flower-beds, and unnecessary woodland have already mostly gone the way such amenities have had to go lately, but where this is not the case it would be easy for the Local Government Board to convey a strong hint to managing committees. Where the area of land at disposal is insufficient, probably neighbouring acres could be acquired. The whole acreage should then be devoted to supplying the requirements of the institution itself by means of the paid employment of ex-patients. Every sanatorium needs vegetables and fruit and eggs and chickens and pork; has a good deal of waste food scraps; and might very well keep horses to draw its coal, fetch its patients, and work on its land. Such agricultural extension of the activities of existing institutions, under existing medical supervision, would better suit with the shortage of money, material, and *personnel* of every description than an ill-starred attempt to set up enough farm colonies to accommodate every consumptive with arrested disease. It would also benefit much our public sanatoria—which have never been in a healthy way—give them stability, and help them to advance to the ordered constitution and management of asylums for the insane, in which employment of patients on the land is a prominent feature.

